# Supplementary material for: The Association between Symptomatic Rotavirus Infection and Histo-Blood Group Antigens in Young Children with Diarrhea in Pretoria, South Africa
Source: Viruses. 2022 Dec 8;14(12):2735. doi: 10.3390/v14122735 (PMC9782691; doi:10.3390/v14122735)
Supplement: Supplementary file 1 [file viruses-14-02735-s001.zip › viruses-1894685-supplementary.pdf]

viruses MDPI

Supplementary

Table S1. Distribution of demographic characteristics in rotavirus positive and negative by site.

	**RV negative n = 293**		**RV positive n = 49**	
	DGMAH	OPHC	DGMAH	OPHC
Patients	72 (24.6%)	221 (75.4%)	29 (59.2%)	20 (40.8%)
Sex				
Male	42 (58.3%)	104 (47.1%)	17 (58.6%)	13 (65%)
Female	30 (41.6%)	117 (52.9%)	12 (41.4%)	7 (35%)
**Ages of the children in months**				
0-6	18 (25%)	65 (29.4%)	6 (20.7%)	4 (20%)
7-12	26 (36.1%)	68 (30.8%)	13 (44.8%)	7 (35%)
13-18	9 (12.5%)	43 (19.5%)	7 (24.1%)	3 (15.0%)
19-24	7 (9.7%)	23 (10.4%)	3 (10.3%)	3 (15.0%)
25-59	12 (16.7%)	22 (9.9%	-	3 (15.0%)
**Rotavirus immunization**				
0 dose (unvaccinated)	2 (2.7%)	2 (0.9%)	-	-
1 dose	8 (11.1%)	10 (4.5%)	4 (13.8%)	1 (5%)
2 doses	49 (68.1%)	196 (88.7%)	23 (79.3%)	15 (75%)
No RTHC[1]	13 (18.1%)	13 (5.9%)	2 (6.9%)	4 (20%)
**Clinical symptoms[2]**				
Fever (n = 334)	47 (65.3%)	20 (9.1%)	17 (58.6%)	3 (15.0%)
Vomiting (n = 332)	26 (36.1%)	23 (10.4%)	16 (55.1%)	5 (25.0%)
Refusal to eat (n = 332)	34 (47.2%)	54 (24.4%)	11 (10.9%)	9 (45.0%)
Duration of diarrhoea days	3.84	4.39	2.86	3.89
